# Study on the Influencing Factors of Miners’ Unsafe Behavior Propagation

**DOI:** 10.3389/fpsyg.2019.02467

**Published:** 2019-11-08

**Authors:** Mengjie You, Shuang Li, Dingwei Li, Qing Xia

**Affiliations:** School of Management, China University of Mining and Technology, Xuzhou, China

**Keywords:** safety atmosphere, safety knowledge, influence degree of key figures, unsafe behavior propagation, mediating effect

## Abstract

This study aims to explore the causes of unsafe behavior propagation (UBP) and then control the spread and prevalence of unsafe behavior in miners’ social networks. Based on social learning theory, this study built a hypothetical model of correlation between safety atmosphere, safety knowledge, influence degree of key figures, and UBP. We administered an empirical study of an effective questionnaire from 433 miners in coal mines via structural equation modeling. The results showed that safety knowledge played a mediating role in the process of UBP influenced by safety atmosphere, and the influence degree of key figures also mediated the relationship between safety knowledge and UBP. Furthermore, the relation between safety atmosphere and UBP was sequentially mediated by safety knowledge and influence degree of key figures. Our research results provided new theoretical and methodological support for intervening in miners’ unsafe behavior.

## Introduction

Research on the unsafe behavior of miners at home and abroad has taken place for more than 70 years. However, studies in recent years have shown that more than 94.09% of mine accidents were attributed to human factors, of which intentional violation, mismanagement and defective design accounted for 35.43, 55.12, 3.54% of cases, respectively (Chen et al., 2012). The reason why the unsafe behavior has not been effectively controlled is that it has the characteristics of transmission, accumulation, extensiveness, uncontrollability, and repeatability. Therefore, how to control the miners’ unsafe behavior propagation (UBP) becomes even more important.

There are widespread transmission phenomena in real life, such as the spread of disease among people (Grassly and Fraser, 2008; Salathé et al., 2010; Goscé et al., 2014), the propagation of computer viruses on the Internet (Zhu et al., 2012; Bonyah et al., 2017; Lazfi et al., 2017), the spread of information and public opinion on various social networks (Wang et al., 2013; Guo et al., 2014; Zhao et al., 2014; Lin et al., 2015), etc., and propagation on complex networks has become a research hotspot of scholars in many fields. Recent studies have shown that besides diseases, information, public opinion and so on can be spread on various networks, and human behavior can also propagate on social networks. Behavior propagation refers to the trend and process of individual behavior triggering the same behavior of neighbors (Wheeler, 1966; Centola, 2010). Centola (2010) pointed out that people’s behavior is similar to information, and in most cases can be spread in the crowd through social contact. When Gordon (1954) put forward his theory of epidemiology, he pointed out that there is a certain similarity between disease and accident, and the occurrence of accidents also has certain infectivity and susceptibility. Subiaul (2010) elaborated the spread of behavior from the perspective of demonstration imitation, pointing out that imitation is the process by which an individual learns how to produce a similar behavior on the basis of observing the demonstrator. Different scholars have carried out in-depth research on behavior propagation. Some studies pointed out that individualization affects the occurrence of behavior propagation (Barsade, 2002), and some studies indicated that social normative pressure limits the occurrence probability of behavior transmission (Gino et al., 2009; Ball et al., 2010), and many other scholars confirmed that behavior propagation is the result of complex effects of multiple influencing factors (Christakis and Fowler, 2013).

In past research, people have paid more attention to the propagation of diseases, information and public opinion, and established various network propagation models based on the average field method (Pastor-Satorras and Vespignani, 2001; Zhou and Liu, 2009; Castellano and Pastor-Satorras, 2010; Garas et al., 2010). However, little research has been done on the behavior propagation on social networks, with most of them being qualitative research, and few quantitative ones. The behavior transmission process in social life is different from epidemic transmission. In the spread of disease, individual contact between network nodes can complete virus transmission under certain probability. While social behavior spread is relatively complicated, it is through individual decision-making whether to accept and join the spread of the process, in which the uncertainty of individual decision-making determines the complexity of social behavior propagation (Acemoglu et al., 2010; Zhang and Wu, 2012; Chen et al., 2015; Zuber, 2015). Therefore, in a large-scale social network, it is of great significance to study the factors that affect individual decision-making in order to control behavioral transmission.

At present, the research on unsafe behavior of miners mainly focuses on exploring the influencing factors of individual unsafe behavior and its occurrence mechanism. From external factors analysis, researchers have demonstrated that safety atmosphere (Neal et al., 2000), leader behavior (Li et al., 2015), organizational policy (Cacciabue and Vella, 2010; Li J.Z. et al., 2017), environment (Papadopoulos et al., 2010), and safety management (Wu et al., 2008) have important implications for employee safety behavior through empirical research. From the analysis of individual internal factors, safety knowledge mastery (Yin et al., 2012), psychological factors (Liu and Luo, 2012), safety skills mastery (Wang L. et al., 2018), and work pressure (Han et al., 2014) have a certain correlation with employee unsafe behavior. Researchers used evolutionary games and other methods to describe the mechanism by which coal mine employees choose unsafe behavior (Meng and Yao, 2011; He et al., 2014; Li J.Z. et al., 2017). Wang X.H. et al. (2018) analyzed the propagation mechanism of UBP among miners based on propagation dynamics theory, and constructed the SIRS propagation model of miners’ UBP. In fact, in the working environment of coal miners in China, most teams are small groups. There is a lot of physical contacts between miners, communicating and transmitting various information and affecting each other’s behavior. Therefore, once the unsafe behavior of coal mine employees is formed, it is easily imitated and learned by other employees and spread under appropriate conditions, so that unsafe behaviors are accumulated and superimposed to eventually induce accidents. Studying the spread of unsafe behavior of miners and predicting, managing and controlling them have important theoretical and practical significance for reducing unsafe behavior of miners.

Unlike the traditional research on the influencing factors and occurrence mechanism of unsafe behavior, the spread of unsafe behavior studied in this paper is a process of copying and emulating unsafe behavior among multiple subjects. The purpose is to study which factors will lead to the spread of unsafe behavior among multiple miners rather than simply exploring which influencing factors can cause individual miners to take unsafe behavior. Therefore, in order to confirm that the unsafe behavior of the miner has occurred, it is caused by the imitation and reproduction of the unsafe behavior of others; this study only considers situations in which miners make the same or similar unsafe behavior after observing the unsafe behavior of others. This paper identifies the influencing factors of miners’ unsafe behavior contagion and puts forward relevant hypotheses through relevant literature research and survey results, and then uses SEM to quantitatively analyze it, which provides a theoretical basis for controlling the spread of unsafe behavior of miners.

## Literature Review and Hypotheses

### Safety Atmosphere and UBP

Zohar (1980) suggested that safety atmosphere is the common cognition of all employees in an enterprise to the dangerous working environment. Mearns and Flin (1999) argued that safety atmosphere is an employee’s perception, belief, and attitude toward risk and safety. At present, in the fields of construction, subway construction, coal mines, etc., scholars have paid attention to the impact of safety atmosphere to unsafe behavior (Cooper and Phillips, 2004; Lin et al., 2008; Martínez-Córcoles et al., 2011; Cheyne et al., 2013; Shin and Kim, 2014; Liu Q. et al., 2015; Liu X. et al., 2015; Toppazzini and Wiener, 2017), but few researchers have focused on the influence of safe atmosphere on UBP. The study found that unsafe behavioral transmission is divided two ways: demonstration imitation and infection conformity (Yu et al., 2015; Han et al., 2016; Zhou, 2016). According to the theory of propagation, the main elements of infection conformity are the safe atmosphere and the psychological state of the behavior recipient (Zhou, 2016; Yang et al., 2018). At the same time, relevant research found that the safety atmosphere of the construction team has a significant effect on the propagation of human unsafe behavior (Han et al., 2016; Cao, 2017; Yang et al., 2018). In modern social psychology, selective imitation refers to imitating people selectively after thinking (Blackmore, 2001). Therefore, when employees are exposed to unsafe behaviors, they will not immediately imitate, but choose whether to spread them through individual decision-making. Employees will improve their safety awareness and attitude in a good safety atmosphere, thereby constraining their own behavior and preventing themselves from imitating and learning from others’ unsafe behavior, thus hindering the spread of unsafe behavior. Therefore, we proposed the following:

**Hypothesis 1:** Safety atmosphere is negatively related to UBP.

### Safety Knowledge and UBP

Safety knowledge is the knowledge and skill for safe operation that operators must possess, including the ability to identify potential safety hazards and make timely decisions in the usual sense (Yu, 2013). Safety knowledge includes safe facts and information, theory and understanding in practice, as well as safety experience, background and awareness gained from education (Fruhen et al., 2014). Safety knowledge is considered to be an important variable affecting employee safety behavior (Campbell, 1996; Yagil, 2000; Choudhry and Fang, 2008; Christian et al., 2009), which has been studied by many scholars, but few scholars have focused on the impact of safety knowledge on UBP. Human behavior can be spread through demonstration imitation (Yu et al., 2015; Han et al., 2016). Modern social psychology divides imitation into adaptive simulation and selective imitation, among which selective imitation refers to imitating people selectively after thinking (Whitehurst, 1977; Blackmore, 2001). Bala and Goyal (1998) argued that when the rewards of different behaviors are unknown, people will use their past experience and the experience of the people around them to guide decisions and choose one of them. That is, when a person is unable to determine the reward of imitating others’ behavior, his own experience and knowledge will largely influence his decision. Rundmo (2000) suggested that employees’ ability to identify risks will act on the choice of unsafe behavior. In discussing the relevance of safety practices and safety behaviors, Vinodkumar and Bhasi (2010) found that employees’ safety knowledge and safety motivations dominate the selection of individual unsafe behaviors. This indicates that in the face of the unsafe behavior of others, his own safety knowledge will affect his value judgment on these unsafe behaviors, and thus he will choose to imitate or not follow these behaviors. Han et al. (2016) and Zhou (2016) found through empirical research on the factors affecting the transmission of unsafe behavior among construction workers that individual safety knowledge can hinder the spread of unsafe behavior. Therefore, when the employee’s safety knowledge is higher, the employee’s ability to identify dangerous behavior and handle hidden dangers is stronger, thus ignoring or even stopping the unsafe behavior of other individual members of the group. Based on this assumption:

**Hypothesis 2:** Safety knowledge is negatively related to UBP.

### Mediating Role of Safety Knowledge

Synthesizing the research results of safety atmosphere at home and abroad, it is generally used to evaluate safety atmosphere from the following dimensions: safety management (Zhang and Zhang, 2007; Pousette et al., 2008), safety training (Zohar, 1980), safety system (Neal et al., 2000), safety regulations (Cox and Cheyne, 2000), and risk perception (Lin et al., 2007; Zhang, 2008). Professional knowledge and skill as well as the ability to identify potential safety hazards and make timely decisions are important factors to measure the level of employees’ safety knowledge (Yu, 2013). Vinodkumar and Bhasi (2010) concluded that safety training can predict safety knowledge and safety motivation through empirical research. Flin et al. (2000) found that employees’ work abilities are related to the safety atmosphere when measuring safety atmosphere. Some scholars have specifically studied the relationship between safety atmosphere and safety behavior and found that safety atmosphere can indirectly affect safety behavior through safety knowledge (Griffin and Neal, 2000; Neal et al., 2000; Seo, 2005). At present, there are few achievements in directly studying the relationship between safety atmosphere and safety knowledge, but some studies have proved that the pre-influence factors of safety atmosphere, such as safety management, safety training, risk perception and so on, will have a positive impact on individual safety knowledge. Therefore, a good safety atmosphere will improve the safety knowledge level of employees. The higher the level of safety knowledge, the less likely it is that the unsafe behavior of others will be imitated (Han et al., 2016), so the safety atmosphere can indirectly hinder the spread of unsafe behavior through safety knowledge. Accordingly, we hypothesize the following:

**Hypothesis 3:** Safety atmosphere is positively related to safety knowledge.

**Hypothesis 4:** Safety knowledge mediates the relation between safety atmosphere and employees’ UBP.

### Influence Degree of Key Figures and UBP

De Tarde (1903), the pioneer of the imitation theory, pointed out in his book “Law of Imitation” that for those of higher status, the people nearest are the most likely to imitate, and that the lower groups tend to imitate the upper groups. Sociologist Lazarsfeld argues that there are public opinion leaders between the mass media and the public, that they are the main source of public access to information and acceptance, and that they spread the mass media’s message to the public as well as publish subjective judgment (Jerabek, 2001). Li Z. et al. (2017) found that the spread of online public opinion is influenced by several influential opinion leaders, and people are more likely to accept information from opinion leaders than to explore the source of information themselves. From the core-edge theory, the more experienced members such as the team leader, technical backbone, and master are more likely to become opinion leaders and act as key demonstrators of unsafe behavior (Yang et al., 2018), and members with relatively low safety knowledge can easily copy their unsafe behavior directly. Generally speaking, managers cannot directly determine the miners’ behavior, but only influence or control the miners’ behavior choice through their own behavior (Cao et al., 2011). Yang et al. (2018) found that the authority and influence of key figures greatly promotes employees to imitate and learn their corresponding unsafe behavior, thereby promoting the spread of unsafe behavior in the study of construction workers’ behavioral propagation. Therefore, when key people produce unsafe behavior, other employees have the possibility to follow their unsafe behaviors, thus allowing unsafe behavior to spread. Conversely, when key personnel conduct safety education for coal mine employees, employees will trust their opinions because of the authority of key people, and give up the choice to imitate and copy the unsafe behavior of other individual members of the group, thus inhibiting the spread of unsafe behavior. So, the influence degree could be positive or negative. This paper only discusses the significant correlation between the influence degree of key people and UBP when the unsafe behavior of key people has occurred. Thus, we propose the following:

**Hypothesis 5:** Influence degree of key figures is positively related to UBP.

### Influence Degree of Key Figures as a Mediator

In order to better understand the influences of key people on the spread of unsafe behaviors, this paper conducted a field interview with a coal mining enterprise in Shandong Province. Interview results showed that there are many formal or informal teacher-apprentice connections among coal miners. Due to the lack of professional skills training institutions, workers’ safety knowledge level is low and they have to rely on imitating or directly copying key persons such as the team leader, technical backbone, and master to acquire skills, the more influential key figures are more susceptible to worker imitation. Numerous studies have shown that safety knowledge has a positive impact on safety behavior (Choudhry and Fang, 2008; Christian et al., 2009). When the safety knowledge of employees is high, the employees’ awareness of work risk and the ability to deal with hidden dangers is stronger. Employees will further judge whether the behavior of key people is safe rather than blindly imitate or directly copy their behavior. Therefore, the higher the safety knowledge of employees, the more safety behavior employees will consciously take. At this time, the influence of key people such as the team leader, technical backbone, and master is weakened. Accordingly, we hypothesize the following:

**Hypothesis 6:** Safety knowledge is negatively related to influence degree of key figures.

**Hypothesis 7:** The relation between safety knowledge and UBP is mediated by influence degree of key figures.

**FIGURE 1:**
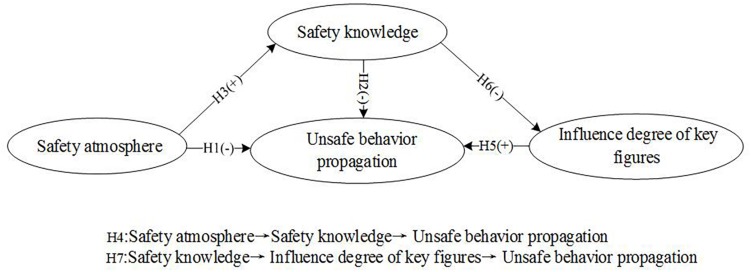
Hypothetical model.

The conceptual model we propose in the present study is depicted in Figure 1.

## Materials and Methods

### Participants and Procedures

#### Participants

The participants of this research are first-line miners from various production departments, such as tunneling, ventilation, comprehensive mining, mechanical and electrical, transportation, and geological surveying, in large state-owned coal mines from Shandong, Henan, Shanxi, Liaoning, Heilongjiang, and other provinces. The data were collected via paper-and-pencil questionnaires. A total of 500 questionnaires were distributed and 451 were finally returned (90.2% response rate). The collected questionnaires were classified and combined with SPSS to draw a box diagram of each variable, find the centrifugal value, and treat the row data of the centrifuged value as invalid data, and delete the sorted invalid data. A total of 433 valid questionnaires were completed, and the effective questionnaire recovery rate was 86.6%.

In this survey, coal mine employees are mainly middle-aged groups. The age groups were as follows: 5.33% aged under 25, 59.56% aged 26–35, 20.10% aged 36–45 years, and 15.01% aged 46 and above. In the distribution of academic qualifications, 39.47% of the participants have senior middle school education, 25.49% of the participants have junior college education, 18.89% of the participants have junior high school and below and 16.16% of the participants have bachelor degree. In the distribution of post-level, the survey targets were mainly rank-and-file employees, with a percentage of 86.41%, first-line managers accounted for 11.41%, middle managers accounted for 2.18%, and no senior leaders accepted the survey. On job tenure, 18.46% had worked for their organization for less than 3 year, 22.54% for 3–5 years, 32.02% for 5–10 years, and 26.98% for more than 10 years. A total of 90.30% of respondents were married, while 7.90% were unmarried and 1.80% reported “other.”

#### Procedures

From March to April 2018, we conducted a large amount of data collection. With the opportunity of national coal mine safety standardization investigation work, we conducted an additional investigation on a total of 500 rank-and-file miners in 20 coal mines. In the process of investigation, each coal mine had a liaison person in charge, and with the assistance of the responsible persons, 25 rank-and-file miners were randomly selected from each coal mine to participate in our investigation. In order to explain the purpose and precautions of the survey to the respondents and to make the participants willing to be truthful, our research team first conducted a brief investigation description with the participants in each coal mine. The interview included the purpose of the survey and showed that the survey did not involve any real names, did not affect the individual, and described some of the considerations in the survey, and we also prepared a beautiful little gift for each participant who completed the survey. After the interview, the survey was conducted in the form of a questionnaire. In order to not delay the participants’ time and ensure the validity of the questionnaire, the participants needed to complete the paper questionnaire within 30 min. The questionnaire was distributed and collected by the research team. The survey was part of a large-scale research project on the behavior of coal miners and relied on the opportunity of national safety standardization investigation to enable the investigation to be completed efficiently and smoothly.

#### Scale Design

In China, the coal mining industry and the construction industry are high-risk industries, and the working environment of workers is based on small groups. Therefore, the observation indicators in the questionnaire of this study mainly refer to the questionnaires of the Chinese scholars Zhou (2016) and Yang et al. (2018) on the factors affecting the transmission of unsafe behavior of construction workers, which have achieved good reliability and validity in the Chinese context. Combining the actual working situation of coal mine enterprises and the management status of unsafe behavior of employees, and referring to many related questionnaires about the influencing factors of unsafe behavior of coal miners, the scale was adjusted and improved to form a preliminary questionnaire on the influencing factors of UBP of coal mine employees.

The questionnaire was designed using the Likert five-point scale method, from 1 (completely disagree) to 5 (completely agree). The content validity of the questionnaire was tested by expert assessment method and on-site pre-test. It was divided into two rounds of expert consultation. In the first round of expert consultation, we invited 10 experts, including 2 rank-and-file miners, 2 team leaders, 2 technical backbones, 1 deputy mine manager, 1 professor engaged in miner behavior research, 1 professor engaged in coal mine safety management research, and 1 master’s degree student familiar with the scale construction process. The 10 experts returned to the revised opinion 2 weeks after receiving the questionnaire, and we modified the questionnaire items according to the comments. The main contents of the revised questionnaire include background information (such as gender, age, type of work, job tenure, education, etc.) and 12 items, which are in a descriptive language that is easy to understand. Three specific items measure safety atmosphere, three specific items measure safety knowledge, three specific items measure influence degree of key figures, and three specific items measure UBP. A second round of expert consultations followed; this time, 10 experts scored the items of the questionnaire through content validity index (CVI). The I-CVI of all items was greater than 0.78 and the K^∗^ was greater than 0.74. Meanwhile, the S-CVI of the scale was 0.94 and greater than 0.9. Therefore, the questionnaire had good content validity. Finally, the questionnaire modified by the expert consultation was pre-tested in one of the research units. Through the analysis of the pre-test questionnaire data, supplemented by literature research and on-site investigation, the final questionnaire was formed. The specific topics of the questionnaire are detailed in Table 1.

**TABLE 1:** Questionnaire items on influencing factors of unsafe behavior dissemination among coal miners.

**Title number**	**Questionnaire items**	**Index**
SA_1	The more often workers around you volunteer to attend safety training and other lectures, the less you will indulge yourself to create unsafe behaviors, thus following the safe behavior of most members.	Safety atmosphere
SA_2	The more strictly the workers around you work in accordance with the operating rules, the less you will indulge yourself to create unsafe behaviors, thus following the safe behavior of most members.	
SA_3	The lower the accident rate and unsafe behavior rate in your mine, the less you will indulge yourself into unsafe behavior and follow the safe behavior of most members.	
SK_1	The higher your skill level, the less likely you are to copy other people’s unsafe behavior.	Safety knowledge
SK_2	The more you value security, the less you will copy the unsafe behavior of others.	
SK_3	The richer your homework experience, the less likely you will copy other people’s unsafe behavior.	
IKF_1	When the team leader has already produced unsafe behavior, the more authoritative the team leader is, the easier it is for you to copy his behavior and produce the same or similar unsafe behavior.	Influence degree of key figures
IKF_2	When a technical backbone has already produced unsafe behavior, the better its professional skills, the easier it is for you to replicate his behavior and produce the same or similar unsafe behavior.	
IKF_3	When a safety pacesetter has already produced unsafe behavior, the better his safe operation habits, the easier it is for you to copy his behavior and produce the same or similar unsafe behavior.	
UBP_1	You will copy the same unsafe behavior based on observing other people’s unsafe behavior.	Unsafe behavior propagation
UBP_2	You will gradually indulge yourself and choose to follow other people’s unsafe behaviors because of a bad safety atmosphere for a long time.	
UBP_3	At work, you are very susceptible to group pressure, resulting in conformity mentality, thus emulating their unsafe behavior.	

#### Data Analysis Strategy

The reliability and validity of the scale data was analyzed using SPSS 23.0. The Cronbach reliability coefficient was used to measure the reliability of each influential item of the sample, and the KMO and Bartlett spherical tests were used to measure whether the sample was suitable for factor analysis. The hypothesis model was tested using the maximum likelihood structural equation model (SEM) with AMOS 22.0. According to the recommendation of Anderson and Gerbing (1988), the two-step method was utilized to test the mediation effects. The first stage was measurement model testing. At this stage, we used confirmatory factor analysis (CFAs; Cheung and Wong, 2011; Choi and Moon, 2017) to test the discriminatory validity of variables. The fit indices of the hypothesis factor model were compared with the alternative factor models to select the optimal model based on the fitness (Mathieu and Farr, 1991; Cheung and Wong, 2011). In the second phase, we compared the fit indices of the proposed model with those of alternative models to determine which model was the best after the first stage verification (Li F. et al., 2013).

In order to study the adequacy of the estimated model, this paper selected χ2/df, root mean square error of approximation (RMSEA), goodness-of-fit index (GFI), comparative fit index (CFI), and normed fit index (NFI) to test the fit of the model. It is acceptable for χ^2^/df to be between one and five (Salisbury et al., 2002). The CFI, NFI, and GFI should be over 0.90 (Salisbury et al., 2002), and the value of RMSEA should be less than 0.08 (Byrne, 2006).

## Results

### Common Method Variance

Common method variance (CMV) refers to the expansion of correlations between variables when collecting data using self-reported questionnaires (Podsakoff et al., 2003). This may lead to false support for hypotheses. To test whether CMV was a problem, we employed Harman’s single-factor test. We loaded all the items of each variable into a factor analysis. The result showed that the first factor explained 20.96% of the variance, which is much less than 50%, indicating that CMV was not a problem in this study.

### Reliability and Validity Analysis

#### Reliability Analysis

To ensure the rigor of the study, the reliability of the questionnaire used in the survey was tested. The specific reliability analysis results are shown in Table 2, which shows that the Cronbach’s Alpha of each variable is greater than 0.7. According to the reliability test criteria, the latent variables of the related topics have high consistency, and the reliability of the questionnaire is acceptable (note: because the theoretical hypotheses are not all positively correlated and belong to different constructs, the reliability coefficient of the total questionnaire is not calculated here).

**TABLE 2:** Results of reliability test.

**Variable**	**Number of questions**	***∂***
Safety atmosphere	3	0.83
Safety knowledge	3	0.86
Influence degree of key figures	3	0.80
Unsafe behavior propagation	3	0.74
		

#### Validity Analysis

To ensure the rigor of the study, the validity of the questionnaire used in the survey was tested. The specific validity analysis results are shown in Table 3. The chi-square value of Bartlett’s sphericity test in the scale is 2416.487 (*p* < 0.0001), so the Bartlett test is significant. The KMO value is 0.806, greater than 0.7, indicating that there is a certain correlation between variables, which is suitable for factor analysis. In addition, using the principal component method to extract the factor, the results show that four common factors with eigenvalue greater than 1 are extracted. Factor rotation adopted the maximum variance method. The cumulative variance interpretation rate of the four factors reaches 61.466%, more than 50%, and all factor loads are above 0.5. The rotated factor structure and the distribution of items are also in line with the theoretical expectations of this study, indicating that the scale has a good structural validity.

**TABLE 3:** Table of factor loading after rotation (influencing factor questionnaire).

**Title number**	**Factor 1**	**Factor 2**	**Factor 3**	**Factor 4**
SA_1	0.795			
SA_2	0.771			
SA_3	0.737			
SK_1		0.822		
SK_2		0.762		
SK_3		0.752		
IKF_1			0.731	
IKF_2			0.716	
IKF_3			0.659	
UBP_1				0.627
UBP_2				0.756
UBP_3				0.791
Variance explanatory volume %	16.955	15.73	14.794	13.987
Cumulative variance interpretation rate	16.955	32.685	47.479	61.466
KMO value			0.806	
Bartlett test chi-square value			2416.487	
*P*-value			0	

#### Measurement Model Testing

In order to test the discriminant validity between key variables, before examining the hypotheses, this study first used AMOS 22.0 to perform confirmatory factor analysis (CFA) on key variables. In order to minimize the magnification of potential variable measurement error, researchers believe that project packages should be created as indicators of variables without sub-scales (Rogers and Schmitt, 2004). Therefore, four latent factors (safety atmosphere, safety knowledge, influence degree of key figures, and UBP) and twelve observed items were contained in the study. The advantages of aggregate-level data (e.g., higher commonality and lower random error) are obvious compared to project-level data (Li M. et al., 2017). The measurement model was tested by comparing the fit indices between the single-factor model (safety atmosphere, safety knowledge, influence degree of key figures, and UBP combined into one factor), 2-factor model (safety atmosphere, safety knowledge, and influence degree of key figures on the same factor; UBP on the other), 3-factor model (safety atmosphere and safety knowledge on the same factor; influence degree of key figures and UBP as separate factors), and 4-factor model (safety atmosphere, safety knowledge, influence degree of key figures, and UBP as separate factors). The results showed that the 4-factor model (χ^2^/df = 1.830, GFI = 0.958, NFI = 0.918, CFI = 0.960, RMSEA = 0.060) had a better fit than the 1-factor, 2-factor, and 3-factor models (see Table 4), so it has a good discriminant validity and can be used for the next SEM analysis.

**TABLE 4:** Comparison of measurement model.

**Structure**	**χ^2^**	**Df**	**χ^2^/Df**	**GFI**	**NFI**	**CFI**	**RMSEA**
1-factor	426.219	35	12.178	0.734	0.367	0.377	0.219
2-factor	267.932	34	7.880	0.814	0.602	0.628	0.172
3-factor	236.756	33	7.174	0.819	0.648	0.676	0.163
4-facotr	54.885	30	1.830	0.958	0.918	0.960	0.060

*1-factor: SA + SK + IKF + UBP; 2-factor: SA + SK + IKF; UBP; 3-factor: SA + SK; IKF, UBP; 4-factor: SA, SK, IKF, UBP. SA, safety atmosphere; SK, safety knowledge; IKF, influence degree of key figures; UBP, unsafe behavior propagation.*

#### Structure Model Testing

**FIGURE 2:**
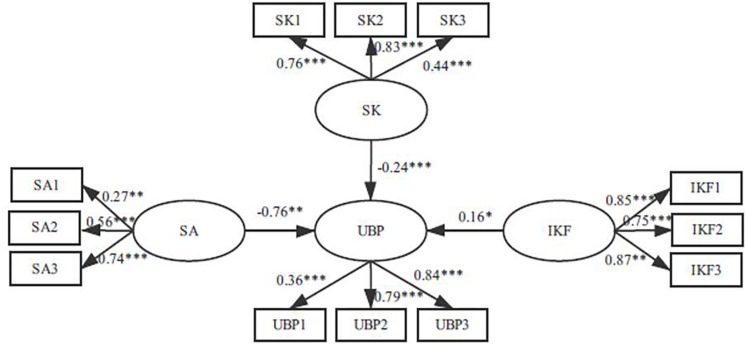
Model 1. SA, safety atmosphere; SK, safety knowledge; IKF, influence degree of key figures; UBP, unsafe behavior propagation; SA1, SA2, and SA3 are three observation variables of safety atmosphere; SK1, SK2, SK3 are three observation variables of safety knowledge; IKF1, IKF2, IKF3 are three observation variables of influence degree of key figures: UBP1, UBP2, UBP3 are three observation variables of unsafe behavior propagation. ^∗^*p* < 0.05, ^∗∗^*p* < 0.01, ^∗∗∗^*p* < 0.001.

This study used SEM to test the mediation effects of safety knowledge and influence degree of key figures. We built several alternative models to test the mediation effects. First, we constructed Model 1 to test the main effects between the various influencing factors and the UBP. In this model, each influencing factor is directly related to UBP (see Figure 2). The result showed that Model 1(χ^2^/df = 2.696, GFI = 0.937, NFI = 0.872, CFI = 0.914, RMSEA = 0.085) did not fit well with the data (see Table 5).

**TABLE 5:** Comparison of the structural models.

**Structure**	**χ^2^**	**Df**	**χ^2^/Df**	**GFI**	**NFI**	**CFI**	**RMSEA**
Model-1	86.262	32	2.696	0.937	0.872	0.914	0.085
Model-2	85.259	31	2.750	0.938	0.873	0.914	0.087
Model-3	54.885	30	1.830	0.958	0.918	0.960	0.060

**FIGURE 3:**
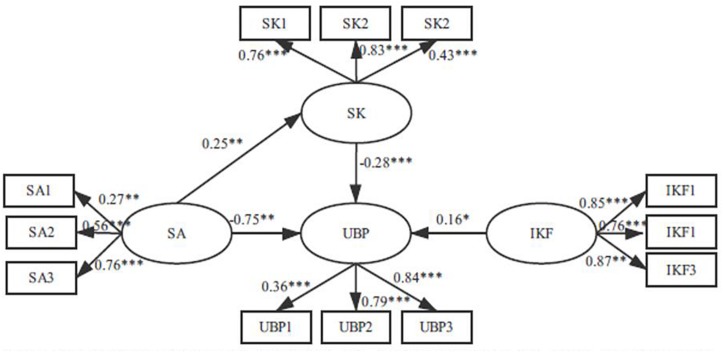
Partially mediated model (Model 2). SA. safety atmosphere; SK, safety knowledge: IKF, influence degree of key figures; UBR, unsafe behavior propagation; SA1, SA2, and SA3 are three observation variables of safety atmosphere; SK1, SK2, SK3 are three observation variables of safety knowledge; IKF1, IKF2, IKF3 are three observation variables of influence degree of key figures; UBP1, UBP2, UBP3 are three observation variables of unsafe behavior propagation. ^∗^*p* < 0.05, ^∗∗^*p* < 0.01, ^∗∗∗^*p* < 0.001.

Second, we added a direct path from safety atmosphere to safety knowledge based on Model 1, thus establishing Model 2 (partial mediation model) (see Figure 3). The results revealed that Model 2 (χ^2^/df = 2.750, GFI = 0.938, NFI = 0.873, CFI = 0.914, RMSEA = 0.087) also has unsatisfactory data fitting. Comparing the path coefficients of Model 1 and Model 2, after adding the mediation variable of safety knowledge, we found that the path coefficient of safety atmosphere to UBP became smaller, indicating that safety knowledge plays a part of the intermediary role between safety atmosphere and UBP. Furthermore, by comparing Model 2 with Model 1, we found that the chi-square difference reached significance, Δχ^2^(1) = 1.002, *p* < 0.05, indicating that Model 2 is better than Model 1 (see Table 5).

**FIGURE 4:**
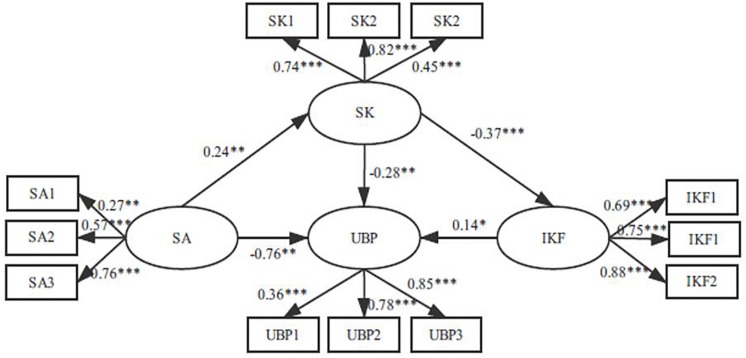
The ultimate mediation model (Model 3). SA, safety atmosphere; SK, safety knowledge; IKE, influence degree of key figures; UBR, unsafe behavior propagation; SA1, SA2, and SA3 are three observation variables of safety atmosphere: SKI, SK2, SK3 are three observation variables of safety knowledge; IKF1, IKF2, IKF3 are three observation variables of influence degree of key figures; UBP1, UBP2, UBP3 are three observation variables of unsafe behavior propagation. ^∗^*p* < 0.05, ^∗∗^*p* < 0.01, ^∗∗∗^*p* < 0.001.

To find the most satisfactory model, we added a direct path from safety knowledge to influence degree of key figures based on Model 2 and built Model 3 (see Figure 4). The results demonstrated that Model 3(χ^2^/df = 1.830, GFI = 0.958, NFI = 0.918, CFI = 0.960, RMSEA = 0.060) fit well with the data. In addition, each latent factor was well represented by its indicators, because factor loadings on these ranged from 0.27 to 0.88 (p < 0.01) (see Figure 3). By comparing Model 3 with Model 2, we found that the chi-square difference [Δχ^2^(1) = 30.374, *p* < 0.001] reached significance, which indicated that Model 3 is superior to Model 2 (see Table 5). Therefore, Model 3 was selected as this study’s final structural model.

According to the recommendation of Preacher and Hayes (2008), we used the bootstrapping method to test the mediation effects displayed in Model 3. Bootstrapping is an ideal way to examine indirect effects, as it avoids non-normal sampling distribution (Zhang et al., 2015). If the 95% confidence interval does not contain zero, then the indirect effects reach a significant level. The results showed that our hypotheses are all verified (see Tables 6, 7 and Figure 4). First, the total effect from safety atmosphere to UBP was notable (β = −0.83, ρ < 0.01), supporting H1. Second, the total effect of safety knowledge on UBP was also significant (β = −0.33, ρ < 0.01), supporting H2. Third, the safety atmosphere had a positive effect on safety knowledge (β = 0.24, ρ < 0.01), and thus H3 was supported. Fourth, the indirect effect of safety atmosphere on UBP via safety knowledge was significant (β = −0.067, ρ < 0.05), supporting H4. Fifth, influence degree of key figures had a positive effect on UBP (β = −0.14, ρ < 0.05), and H5 was confirmed. Sixth, the path coefficient between safety knowledge and influence degree of key figures was notable (β = −0.37, ρ < 0.001), confirming H6. Seventh, the indirect effect from safety knowledge to UBP via influence degree of key figures was significant (β = −0.052, ρ < 0.05), supporting H7. Finally, we also proved that the link between safety atmosphere and UBP was sequentially mediated by safety knowledge and influence degree of key figures (β = −0.012, ρ < 0.05).

**TABLE 6:** The result of the study.

**Hypothesis**	**Estimate effect**	**Get supported or not**
H1	–0.83^∗∗^	YES
H2	–0.33^∗∗^	YES
H3	0.24^∗∗^	YES
H4	−0.067^∗^	YES
H5	0.14^∗^	YES
H6	–0.37^∗∗∗^	YES
H7	−0.052^∗^	YES

*^∗^p < 0.05, ^∗∗^p < 0.01, ^∗∗∗^p < 0.001.*

**TABLE 7:** Direct and indirect effects and 95% confidence intervals in final model 3.

**Model pathways**	**Estimated effect**	**95%CI**	
		
		**Lower bounds**	**Upper bounds**
**Total effect**			
SA-UBP	–0.83^∗∗^	–0.981	–0.679
SK-UBP	–0.33^∗∗^	–0.522	–0.183
**Direct effects**			
SA-UBP	–0.76^∗∗^	–0.894	–0.626
SA-SK	0.24^∗∗^	0.056	0.424
SK-UBP	–0.28^∗∗^	–0.537	–0.023
SK-IKF	–0.37^∗∗∗^	–0.625	–0.115
IKF-UBP	0.14^∗^	0.004	0.276
**Indirect effects**			
SA-SK-UBP	−0.067^∗^	–0.086	–0.048
SK-IKF-UBP	−0.052^∗^	–0.097	–0.007
SA-SK-IKF-UBP	−0.012^∗^	–0.019	–0.005

*SA, safety atmosphere; SK, safety knowledge; IKF, influence degree of key figures; UBP, unsafe behavior propagation. ^∗^p < 0.05, ^∗∗^p < 0.01, ^∗∗∗^p < 0.001.*

## Discussion

Miners’ unsafe behavior will directly or indirectly cause losses to coal mine organizations (Li et al., 2016). There are many studies on the causes and consequences of unsafe behavior of miners, but there are few studies on the spread of unsafe behavior among miners. In this study, through the analysis and comparison of hypothesis model and substitution model, we found that safety atmosphere and safety knowledge are negatively correlated with UBP, and the relationship between safety atmosphere and UBP is partly mediated by safety knowledge; the influence degree of key figures is positively correlated with UBP, and the relationship between safety knowledge and UBP is partly mediated by the influence degree of key figures.

### Theoretical Implications

Our research has some theoretical significance. Firstly, we used the relevant theories of propagation for reference and explored the role of behavioral propagation in the process of miners’ unsafe behavior through empirical research. Previous studies on controlling miners’ unsafe behavior often focused on reducing the occurrence of unsafe behavior by improving monitoring methods (Chen and Liu, 2011; Li S. et al., 2013) and seldom studied the mechanism of multiple factors influencing miners’ unsafe behavior from the perspective of the spread of unsafe behavior. Therefore, this study goes beyond the limitations of previous studies, helps to deepen the understanding of the role of behavioral propagation in the process of miners’ unsafe behaviors, and provides a basis for curbing the occurrence of miners’ unsafe behaviors from the perspective of behavioral propagation. In addition, this study comprehensively considers the influence of internal (safety knowledge) and external factors (safety atmosphere and the influence degree of key figures) on the spread of unsafe behaviors of miners, which provides ideas for further research on the propagation of unsafe behaviors of miners.

Secondly, this paper tests the role of safety atmosphere, safety knowledge, and the influence degree of key figures in the propagation of miners’ unsafe behavior. Through empirical research, we find that safety atmosphere not only directly affects safety knowledge and UBP but also affects UBP through the mediation effect of safety knowledge and the influence degree of key figures. At the same time, safety knowledge not only directly affects the influence degree of key figures and UBP but also affects UBP through the mediation effect of the influence degree of key figures. This indicates that the internal factors of the miners and the external factors of their environment will affect the spread of their unsafe behaviors. In the process of the propagation of unsafe behavior of miners, the factors are not independent individuals; this helps us understand the mode of action among the factors affecting the spread of unsafe behaviors among miners and provides ideas for the study of reducing unsafe behaviors by inhibiting the path of spreading unsafe behaviors.

### Practical Implications

The research results have practical significance for controlling the unsafe behavior of miners from the perspective of behavioral propagation. First, considering that the safety atmosphere can not only directly affect the spread of unsafe behavior but also have an impact on safety knowledge and the influence of key figures, it is necessary to pay attention to the influence of safety atmosphere in the process of the propagation of unsafe behavior of miners. To this end, the organization should start from the perspective of changing leadership style (Wu et al., 2008), shaping organizational culture (Hartmann et al., 2009) and improving the responsibility system to create a good safety atmosphere for miners. In practical situations, it is unrealistic to adjust leadership style to meet the need of shaping a safe atmosphere at any time. In this case, organizations should focus on shaping organizational culture and improving the responsibility system. Managers should actively create an organizational environment full of trust and take their actions as an example to mobilize employees to shape an organizational culture that attaches importance to safety (Kane-Urrabazo, 2006). In addition, the organization should establish and improve the responsibility system of the coal mine, clarify the responsibilities of each employee in order to eliminate the responsibility shifting among the members of the organization, and promote the miners to spontaneously create a good safety atmosphere in the coal mine.

Second, because safety knowledge is negatively correlated with the influence of key people and UBP, miners’ safety knowledge should play a positive role in the process of suppressing the spread of unsafe behavior. Organizations can improve the overall safety knowledge level of miners in various ways, such as shaping a good organizational culture mentioned above, which can provide internal motivation for miners to actively improve safety knowledge level (Dubey et al., 2017). In addition, the organization should strengthen the training and assessment of miners’ safety knowledge, and the training and assessment of safety knowledge should not be superficial. The organization can build a model base of miners’ quality according to its own ability, clarify the safety knowledge needed by miners in various positions, and record the safety knowledge level of each miner and the results of previous safety knowledge assessment, and then dynamically determine who needs to be trained according to the information of the model base, and formulate a training and assessment plan for each miner dynamically.

Finally, in order to reduce the promotive effect of the influence of key figures on the spread of unsafe behavior of miners, the organization should provide enough correct safety behavior hints for miners to weaken the influence of key people on unsafe behavior of general miners. Organizations can place safety behavior tips in operation areas, high risk areas, and personnel-intensive areas to provide correct operation guidance for miners at any time, so as to reduce the dependence of inexperienced miners on the unsafe experience of key persons such as team leaders, thereby eliminating the adverse effects of key persons.

### Limitations and Future Research

Inevitably, this research has some limitations. First, our study uses cross-sectional design. Therefore, it would be premature to draw exact conclusions about causality. For example, in our study, we hypothesized that miners with adequate safety knowledge are less vulnerable to the impact of key people on their unsafe behavior. However, the causality may also be that due to the influence of key people, miners use the irregular experience from key people to replace the safety knowledge that should be acquired through safety training, resulting in a low level of safety knowledge. Therefore, in order to further confirm the causal relationship between factors, future research should adopt longitudinal and experimental research methods.

Second, we use personal questionnaires to evaluate variables. Because of the social desirability response bias, respondents may conceal their true thoughts to some extent (Arnold and Feldman, 1981); this may lead to some deficiencies in the results of the questionnaire survey. Therefore, we should introduce other measurement methods to our future research, such as colleague assessment, leadership assessment, and behavior observation and so on, to enhance the effectiveness of the survey results.

Last, since the data in this study were derived from a questionnaire survey of miners in several large coal mines in several provinces of China, and there is no survey of small and medium-sized coal mines, the miners surveyed may not truly represent all the coal miners in China. In addition, different industries may have different HR practices and organizational cultures, and the same research may lead to different conclusions in different industries. In order to make our research universal, future researchers should test our models in more industries to extend the research conclusions.

## Data Availability Statement

The datasets generated for this study are available on request to the corresponding author.

## Ethics Statement

This study was carried out in accordance with the recommendations of ethics committee of China University of Mining and Technology with written informed consent from all subjects. All subjects gave written informed consent in accordance with the Declaration of Helsinki. The protocol was approved by the ethics committee of China University of Mining and Technology.

## Author Contributions

MY designed and drafted the work. SL collected the data. DL revised the manuscript. QX analyzed data for the study.

## Conflict of Interest

The authors declare that the research was conducted in the absence of any commercial or financial relationships that could be construed as a potential conflict of interest.
